# Effects of Rootstock and Exogenous Plant Growth Regulators on Volatile Aroma Profiles and Terpenoid-Mediated Defense in Table Grape Fruit

**DOI:** 10.3390/life16040567

**Published:** 2026-03-31

**Authors:** Yuyang Zhao, Tao Xu, Lingling Hu, Yanfei Guo, Zhihui Chen, Yueyan Wu, Zhongyi Yang

**Affiliations:** 1 College of Biology and Environment, Zhejiang Wanli University, Ningbo 315000, China; 2 College of Life Sciences, Dundee University, Dundee DD1 4HN, UK

**Keywords:** grape, aroma, rootstock, GA3, MeJ, Terpenoids, antifungal activity

## Abstract

The aroma quality of grape fruit is a crucial trait for table grapes, yet its relationship with plant disease resistance remains unclear. Using ‘Shine Muscat’ grapes as material, this study employed HS-SPME-GC-MS combined with odor activity value (OAV) and PLS-DA analysis to investigate the regulatory effects of different rootstocks and GA_3_/MeJA treatments on volatile aroma compounds. Linalool and α-terpineol were selected as representative compounds for antibacterial experiments and gene expression analysis of terpenoid synthesis. Results indicate that the Lot rootstock and 15.25 mg·L^−1^ GA_3_ treatment significantly promoted the accumulation of terpenoid aroma compounds. Linalool exhibited significant inhibitory effects on the mycelial growth of *Colletotrichum fructicola* and induced upregulation of *DXS*, *TPS56*, and *TPS* gene expression. This study reveals a potential link between aroma metabolism and defense responses, providing a theoretical basis for synergistic optimization of grape aroma quality improvement and disease-resistant cultivation.

## 1. Introduction

Aroma is one of the core traits determining the sensory quality and market value of grapes (*Vitis vinifera* L.). determining the sensory quality and market value of both table grapes and wine products [1]. Its complexity stems from the synergistic interaction of multiple classes of volatile organic compounds (VOCs) [2]. Hundreds of VOCs have been identified in grape fruit, primarily including terpenes, alcohols, aldehydes, esters, and C6 and C9 compounds derived from the lipoxygenase pathway. The contribution of these compounds to fruit aroma varies significantly due to differences in molecular structure and odor thresholds, which may vary depending on the food matrix in which they are measured [3]. Research indicates that even trace concentrations of certain volatile compounds can strongly influence aroma perception due to their extremely low odor thresholds reported in model or aqueous systems, highlighting the limitations of evaluating aroma quality solely based on total volatile content [4]. In Muscat-type grapes, terpenoid compounds are considered key substances forming typical floral and fruity characteristics, with their accumulation levels and compositional ratios directly determining fruit aroma [5]. Furthermore, grape aroma characteristics are not only controlled by varietal genetic backgrounds but are also significantly regulated by cultivation practices and environmental conditions, offering substantial potential for improving fruit aroma through agricultural management strategies [6].

Among various cultivation control factors, rootstock grafting is widely applied in grape production systems. Initially employed primarily to enhance plant stress tolerance, regulate growth vigor, and improve yield stability, its impact on fruit quality and aromatic characteristics has gradually gained attention in recent years [7]. Previous studies have demonstrated that different rootstocks significantly influence the composition and content of volatiles in fruit by affecting water and mineral nutrient uptake capacity, regulating plant growth balance, and altering fruit ripening processes [8]. Among varieties such as Cabernet Sauvignon and Chardonnay, different rootstocks can significantly alter the proportions of alcohols, aldehydes, and terpenes in the fruit, thereby shaping distinct aromatic profiles [9]. Research on the table grape ‘Shine Muscat’ also indicates that rootstock variation not only affects fruit soluble solids and acidity but also significantly regulates the accumulation levels of monoterpenes and C6 volatiles, thereby influencing fruit aroma expression [10]. Furthermore, results from multi-year, multi-location trials indicate that certain vigorous rootstocks may increase C6 alcohol content in fruit, enhancing grassy or green characteristics, while moderately vigorous rootstocks better facilitate the balanced expression of fruity and floral aromas [11]. Nevertheless, systematic comparisons of the effects of commonly used rootstocks on the aroma of table grapes under identical ecological conditions remain limited, and conclusions in this area are inconsistent.

Beyond rootstock selection, exogenous plant growth regulators also play a crucial role in regulating grape fruit development and quality formation. Among these, gibberellic acid (GA3) and methyl jasmonate (MeJA) are the two most widely used types of regulators in production [12]. GA3 is commonly used to promote fruit enlargement and alter fruit shape. Its application timing and dosage can significantly influence the fruit ripening process and the accumulation of various primary and secondary metabolites [13]. Previous studies have demonstrated that GA3 treatment not only alters fruit morphological traits but also indirectly regulates the content of certain volatile aroma compounds in fruit by influencing ripening rate and carbon metabolism allocation [12]. In contrast, as a plant stress and defense signaling molecule, MeJA exhibits a more direct role in regulating secondary metabolism and aroma formation. Studies have shown that exogenous application of this compound can increase the accumulation of terpenoids and esters in Sangiovese grapes by approximately twofold [14]. Field application of MeJA significantly enhances fruit aroma intensity in Tempranillo and other grape varieties, but its regulatory effects show marked variations across different vintages and cultivation conditions [15]. However, current research on GA3 and MeJA affecting the aroma of table grapes has primarily focused on single-factor analyses. The combined effects of different hormones on aroma formation under specific rootstock backgrounds remain understudied [16].

Terpenoids are not only key components of fruit aroma but also crucial secondary metabolites in the plant defense system [17]. Previous studies have demonstrated that monoterpenes such as linalool and α-pinene exhibit inhibitory effects against various plant pathogenic fungi [18], and can affect cell membrane integrity [19,20] or induce the expression of defense-related genes involved in plant-pathogen interactions. These studies confirm the vital role of terpenoids in plant antimicrobial defense at the levels of chemical ecology and molecular biology, providing crucial evidence for understanding the biological significance of aromatic metabolism. However, existing research has primarily focused on the antibacterial activity of individual compounds, with insufficient attention given to whether changes in terpene accumulation induced by fruit aroma regulation measures (such as rootstock grafting and exogenous plant growth regulators) simultaneously affect plant disease resistance. The intrinsic link between aroma quality formation and defense responses remains lacking in systematic experimental evidence. To address this gap, this study analyzed the accumulation patterns of terpenoid aroma compounds in grape fruits following rootstock and GA_3_/MeJA treatments. Using linalool and α-terpineol as representative compounds, antibacterial experiments were conducted. Combined with expression analysis of key terpenoid synthesis genes *DXS*, *TPS*, and *TPS56*, suggesting a potential association between terpene metabolism and defense-related responses. This provides new theoretical foundations for synergistically optimizing grape aroma quality improvement and disease-resistant cultivation.

## 2. Materials and Methods

### 2.1. Fruit Materials and Treatments

All experimental materials were collected from five-year-old ‘Shine Muscat’ grapevines grafted onto different rootstocks at the Shengpu vineyard in Cixi, Zhejiang, China. (30°16′6.59″ N, 121°25′2.18″ E). The rootstocks tested included three domestic cultivars—Kangzhen No.1 (K1), Kangzhen No.3 (K3), and Kangzhen No.5 (K5)—developed by the Zhengzhou Fruit Research Institute of the Chinese Academy of Agricultural Sciences, along with eight introduced rootstock cultivars: 1103 Paulsen (1103P), 3309 Millardet (3309M), S04, 110 Richter (110R), Telekki 8B (8B), Millardet Grasset 101-14 (101-14), Rupestris du Lot (Lot), and Couderc 161-49 (161-49). The vineyard has an average annual temperature of 16.0 °C, average precipitation of 1272.8 mm, and an average yearly sunshine duration of 2038 h. Grafting was done in May 2019, and the vines started bearing fruit in 2021. For this experiment, grapevines with uniform vigor, canopy size, and growth status were selected. The entire vineyard was managed under consistent conditions, using an east–west oriented steel-framed rain-shelter system with continuous-span greenhouses. Vines were spaced at 2 m × 3 m, trained on a Y-shaped trellis system, with a trunk height of approximately 1.5 m [21]. Growth performance across the ‘Shine Muscat’ vines grafted onto different rootstocks was generally stable and healthy.

Grape clusters were soaked for 5 min at 15 days after full bloom in two gibberellic acid (GA_3_) solutions: 22.5 mg L^−1^ (C1) and 15.25 mg L^−1^ (Z1). In addition, two methyl jasmonate (MeJA) treatments were applied by spraying grape clusters at the second stage of berry expansion with 1500 μmol L^−1^ and 2000 μmol L^−1^ MeJA solutions, respectively. After spraying, the clusters were bagged for three days. Control treatments included clusters soaked in distilled water for 5 min at 15 days after full bloom, and spraying during the second berry expansion stage was also used as a control. Detailed treatment protocols are provided in Table 1.

Three biological replicates were collected for each treatment group, with three grape clusters per replicate. From each cluster, berries were evenly sampled from the top, middle, and bottom sections, resulting in a total of 15 berries per treatment. The berries were randomly chosen for further analysis. Fresh berries were weighed immediately and used to measure volatile aroma compounds. The remaining berries were quickly frozen in liquid nitrogen and stored at −80 °C for later analyses.

### 2.2. HS-SPME-GC-MS Analysis

Extraction and analysis of aromatic compounds were carried out as described in [1] with slight modifications. A total of 50 g of berries was ground in liquid nitrogen, centrifuged, and filtered to obtain a clear juice. Grape juice (6 mL) was transferred to a 20 mL glass vial (Agilent Technologies, Santa Clara, CA, USA) containing 1.5 g NaCl. 2-Octanol was added to the pulp juice as an internal standard to quantify aroma compounds. The sample vial was equilibrated at 50 °C for 10 min. Then, solid phase microextraction fiber (SPME, 50/30 μm DVB/CAR/PDMS, Supelco, Bellefonte, PA, USA) was used to extract volatile aroma compounds at 50 °C for 30 min. The fiber was then immediately inserted into the gas chromatograph (Agilent 8890 GC, Agilent Technologies) injection port for desorption at 260 °C for 3 min in the splitless mode, and it was equipped with a 5977 mass-selective detector (Agilent Technologies). The volatile aroma compounds were separated using an HP-Innowax column (30 mm × 0.25 mm × 0.25 μm; Agilent Technologies). The temperature program was as follows: 40 °C for 5 min, increased to 240 °C at a rate of 5 °C/min and then increased to 260 °C at 20 °C/min before being held for 5 min. The flow rate of helium as a carrier gas was 1 mL/min. The mass spectrometry (MS) transfer line and ionization source temperature were 260 °C and 230 °C, respectively. Electron impact mass spectrometric data from 20–400 m/z were obtained at 70 eV ionization voltage.

The volatile aroma compounds were identified by comparing their mass spectra with those from the NIST14 mass spectral library. Only compounds with a mass spectral match percentage greater than 80% were selected for further analysis. The quantification of volatile compounds was performed using a semi-quantitative method. The concentration of each compound was calculated based on the internal standard (2-octanol), assuming a response factor of 1. The concentrations of the volatile aroma compounds were estimated using the following method:

CSC=ASCAIS×CIS×VISVS

where A_SC_ is the peak area of a specific compound, A_IS_ is the peak area of an internal standard, C_SC_ is the concentration of a particular compound, C_IS_ is the concentration of the internal standard, vs. is the volume of samples, and V_IS_ is the volume of the internal standard.

### 2.3. Odor Activity Value (OAV) Calculation

The odor activity value (OAV) was used to evaluate the contribution of individual volatile compounds to the overall aroma profile. The OAV was calculated using the formula:

$$OAV=\frac{C_{i}}{{OT}_{i}}$$

where C_i_ is the concentration of the specific volatile compound in the sample, and OT_i_ is its corresponding odor threshold in water or a similar matrix.

### 2.4. Mycelial Growth Inhibition Test

The *Colletotrichum fructicola* strain was isolated from grape fruits. Conidia were obtained by incubating this strain on potato dextrose agar (PDA) medium at 28 °C for 7 days. Spores were briefly frozen in liquid nitrogen and then ground into a fine powder using a sterile mortar and pestle. Total RNA was extracted using the Spin Column Fungal Total RNA Purification Kit (Sangon Biotech, Shanghai, China) and subsequently reverse transcribed into cDNA. Internal transcribed spacer (*ITS*), actin (*ACT*), and glyceraldehyde-3-phosphate dehydrogenase (*GAPDH*) genes were amplified using specific primers. The primers are shown in Table S1.

α-Terpineol (chemically synthesized, analytical grade, purity 98%) was dissolved in dimethyl sulfoxide (DMSO) and added to PDA medium to prepare a 225 μL L^−1^ α-Terpineol solution. An equal volume of DMSO solution was added as a control. The final DMSO concentration did not exceed 0.1%, and a DMSO-only treatment served as the control. A 9 mm diameter agar plug containing mycelium was excised from the edge of a vigorously growing *Colletotrichum fructicola* colony and placed at the center of the medium plate. Plates were incubated for 7 days (28 °C dark incubation). Ten replicates were set up for each treatment group. Antifungal activity was assessed by measuring colony diameter using a cross-sectional method.

The linalool antimicrobial assay was adapted from the method described by Hong et al. [20]. In brief, Linalool (chemically synthesized, analytical grade, 98% purity) was diluted in anhydrous ethanol to a 20% (*v*/*v*) solution. A 25 μL drop of this diluted solution was applied onto a square sterile paper disc (10 mm edge length) moistened with 15 μL of anhydrous ethanol, ensuring the disc contained 5 μL of Linalool. Forty μL of 95% ethanol was dropped onto a paper disc as an untreated control. The paper discs were air-dried for 20 min at room temperature under sterile conditions to evaporate ethanol until used.

### 2.5. Leaf Infection and qRT-PCR

Fresh ‘Shine Muscat’ leaves were collected and inoculated with *Colletotrichum fructicola* fungal blocks containing PDA medium at the leaf center, with PDA medium blocks serving as controls. The inoculation concentration is 1 × 10^7^ conidia/mL. After 7 days of incubation (28 °C with 16 h photoperiod), healthy and infected leaf sections were harvested. The total RNA was extracted using the HipPure Plant RNA Mini Kit (Magen, Guangzhou, China) and first-strand cDNA was synthesized using First-Strand cDNA Synthesis SuperMix (Novoprotein, Suzhou, China). Three genes, including *VvDXS* (VIT_05s0020g02130) [22], *VvTPS56* (VIT_10s0003g00890) [23], *VvTPS* (VIT_02s0025g04090) [24]. Perform qRT-PCR using the TransStart Tip Green qPCR Super rMixS kit (Novoprotein, China) and q225 fluorescence quantitative PCR instrument (Kubo, Guangzhou, China). The relative expression levels of target genes were calculated using the 2^−ΔΔCT^ method. The qRT-PCR primers are shown in Table S1.

### 2.6. Statistical Analysis

Each treatment was set up with 3 biological replicates, each replicate comprising 3 ears. Five randomly selected kernels from each ear were pooled to form a single sample for GC-MS analysis. Each sample underwent 3 technical replicates. Statistical analysis was performed using biological replicates as the experimental unit (*n* = 3). The heatmap was generated using TBtools-II v2.330. Principal component analysis (PCA) and orthogonal partial least squares discriminant analysis (PLS-DA) were carried out with SIMCA 14.1 (Umetrics, Umeå, Sweden) for multivariate data analysis, result extraction, and visualization. Variable importance in projection (VIP) scores were obtained from the PLS-DA models. Statistical analysis and visualization were performed using SPSS 27.0.1.0 (IBM Corp., Armonk, NY, USA) and GraphPad Prism 9 (Harvey Motulsky, Santiago, CA, USA) software. Following one-way analysis of variance (ANOVA), Tukey’s test or Duncan’s test was applied. Statistical significance was determined using Tukey’s HSD (Honestly Significant Difference) test for multiple comparisons. Graphical illustrations were finalized using Adobe Illustrator 2021 (Adobe Systems Inc., San Jose, CA, USA).

## 3. Results

### 3.1. Rootstock Grafting Treatment

#### 3.1.1. Effects of Different Rootstocks on Fruit Morphology of ‘Shine Muscat’

**Figure 1:**
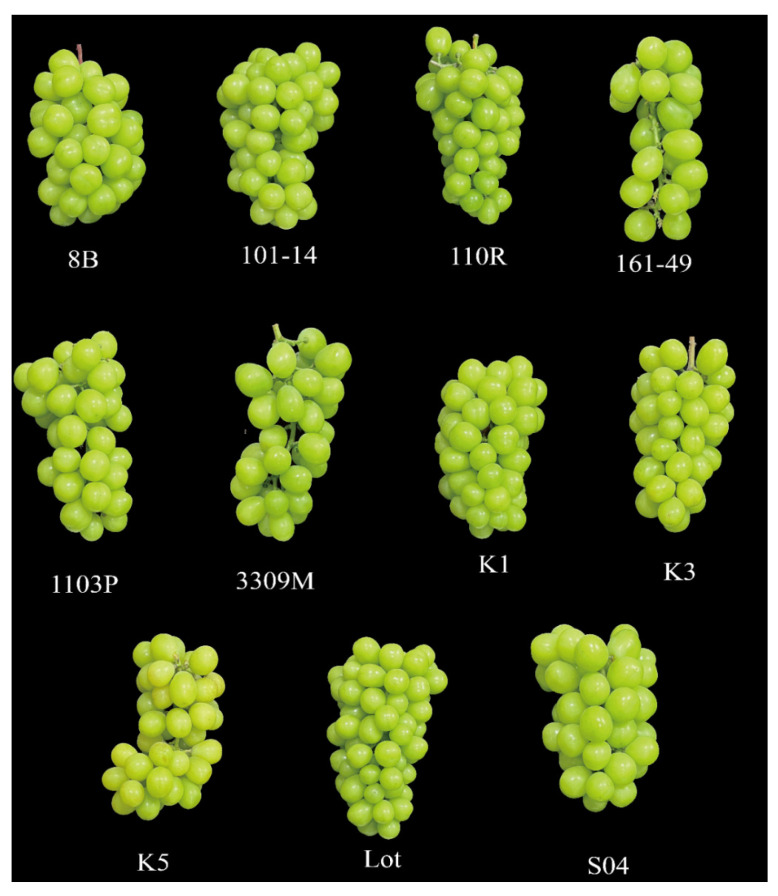
Fruit morphology after grafting on different rootstocks. Fruit was harvested from 5-year-old vines grafted in 2019, and three bunches of ripe grape fruit with uniform growth conditions were picked from each treatment group.

To evaluate the effects of different rootstocks on fruit characteristics and aroma metabolism in ‘Shine Muscat’ grapes, a systematic analysis was conducted on the appearance traits and volatile organic compound (VOC) composition of mature fruits grafted onto 11 commonly used rootstocks. Under different rootstock treatments, variations were observed in cluster compactness, berry size, and fruit uniformity (Figure 1). Specifically, fruits grafted onto Lot, 8B, and SO4 rootstocks exhibited larger berries and relatively consistent berry shape, whereas those on 161-49 rootstock produced looser clusters with significant berry size variation.

Although rootstocks exert some influence on fruit morphology, this study focuses on their regulatory role in the accumulation of aroma-related volatile compounds in fruit. Subsequent analyses primarily examine the compositional characteristics of fruit volatiles and their contribution to aroma under different rootstock conditions.

#### 3.1.2. Effects of Different Rootstocks on the Composition Characteristics of Fruit Volatile Compounds

**Figure 2:**
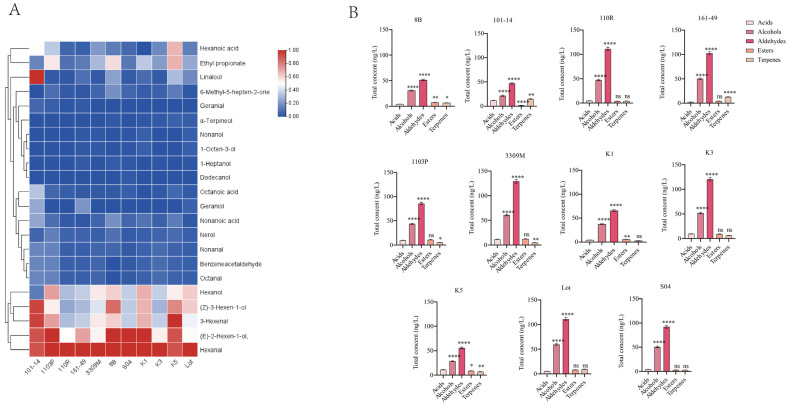
(**A**) GC-MS results of rootstock-grafted fruits heat map. The Normalization Method, Col scale and Cluster Rows were used for preparing the heatmaps. Blue to red indicated a gradual increase in concentration. (**B**) Comparison of the relative content of volatile substance categories in grafted grape berries on different rootstocks. Vertical bars indicate mean ± SD (*n* = 3), and different * indicate significant differences between different kinds of substances (one-way ANOVA, *p* < 0.05) and “ns” statistically significant difference (*p* > 0.05).

Based on HS-SPME–GC–MS analysis, the volatile organic compound (VOC) profiles of mature ‘Shine Muscat’ grape fruits under different rootstock grafting conditions were systematically compared. Twenty-two common volatile compounds were detected across 11 rootstock treatments, primarily comprising aldehydes, alcohols, terpenes, acids, esters, and ketones (Figure 2A). Overall, the types of volatiles in fruits were relatively consistent across rootstock treatments, but significant differences existed in the relative abundance and accumulation levels of various compounds. Aldehydes and alcohols consistently dominated as the major components of fruit volatiles. Results indicate that the lipoxygenase-related metabolic pathway maintained high activity during the ripening stage of ‘Shine Muscat’ fruit. However, different rootstocks may influence the metabolic flux of this pathway, leading to variations in the content of C6 compounds.

Among all rootstock treatments, aldehydes and alcohols consistently dominated the volatile compounds in the fruit (Figure 2B). Among these, C6 aldehydes and C6 alcohols, such as hexanal, (Z)-3-hexenal, (E)-2-hexen-1-ol, and (Z)-3-hexen-1-ol, exhibited high accumulation levels under different rootstock conditions. This indicates that the lipoxygenase pathway-related metabolism plays a fundamental role in the formation of ‘Shine Muscat’ fruit aroma.

Comparative results across rootstocks indicate relatively higher accumulation levels of C6 aldehydes and alcohols in fruits grown on rootstocks such as K3, Lot, and 8B, whereas rootstocks 101-14 and 110R exhibited comparatively lower levels of these compounds (Figure 2A). Significant differences in the accumulation levels of C6 aldehydes and alcohols were observed across rootstock conditions, indicating a strong association between rootstock type and the relative abundance of lipid oxidation-related volatile compounds.

Compared to aldehydes and alcohols, acid compounds exhibited a lower proportion across all rootstock treatments, with smaller overall variations in content (Figure 2B). The detected acids primarily included hexanoic acid, octanoic acid, and nonanoic acid. Among these, hexanoic acid exhibited relatively high levels under rootstocks K5, 101-14, and 1103P, while the lowest levels were observed under rootstocks K1 and 161-49. The regulation of acid volatile accumulation levels by different rootstocks resulted in variations in the relative content of acid volatiles across rootstock treatments.

Although terpenoids constitute a relatively low proportion of total volatile compounds, their variations among different rootstocks are quite pronounced. Aromatic monoterpenes such as linalool, geraniol, and α-terpineol showed a marked enrichment trend in certain rootstock treatments, particularly under 101-14, 8B, and Lot rootstocks, where their relative content exceeded that of other rootstocks. Terpenoids typically originate from the methyl erythritol phosphate (MEP) pathway and serve as the fundamental compounds responsible for the floral and fruity characteristics of Muscat-type grapes. Despite their low absolute concentrations, their extremely low olfactory thresholds create an amplifying effect in fruit aromas, making their variations crucially influential in fruit fragrance profiles.

Regarding esters and ketones, fewer types were detected across rootstock treatments, with overall content remaining at low levels. Ethyl propionate exhibited relatively higher levels under rootstocks 1103P, 8B, and K5 (Figure 2A), while showing lower content in other rootstock treatments. The generally low ester content observed in this study may be related to the volatile metabolite characteristics of ‘Shine Muscat’ during ripening.

A comprehensive analysis of the category composition and relative content changes in volatile compounds under different rootstock conditions reveals that rootstock did not alter the fundamental composition types of volatile compounds in ‘Shine Muscat’ fruit. Instead, it primarily reshaped the proportional structure of volatile compounds by regulating the accumulation levels of various volatile compounds. In particular, the differential regulation of fundamental aroma components such as aldehydes and alcohols, along with key aromatic compounds like terpenes, lays the foundation for subsequent aroma contribution analysis based on odor activity value (OAV) and rootstock selection.

#### 3.1.3. OAV Analysis Reveals Differences in Aroma Contributions Among Different Rootstocks

**Figure 3:**
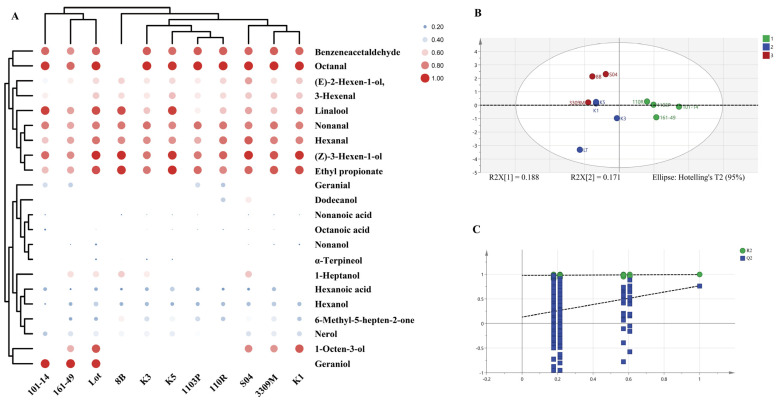
Clustered heatmap of aroma OAV in rootstock-grafted fruits. (**A**) The log2-transformed monoterpenes values were used for preparing the heatmaps. Blue to red indicated a gradual increase in monoterpenes concentration. Hierarchical clustering of VOCs in OAV analysis of differential aroma using the PLS-DA analysis. (**B**) PLS-DA score plot for classification; The PLS-DA model achieved an R^2^X (cumulative explained variance of predictors) of 0.94, an R^2^Y (cumulative explained variance of the response) of 0.991, and a Q^2^ (predictive ability) of 0.661. (**C**) model cross-validation results. The model includes 200 displacement tests. The data for PLS-DA was processed using only unit variance and was not log-transformed.

Since the sensory contribution of volatile compounds depends not only on their absolute content but also closely relates to their olfactory threshold, an odor activity value (OAV) analysis was further conducted on the detected volatiles (Figure 3A, Table S2). Results revealed that among the 22 detected VOCs, multiple aldehydes, terpenes, and C6 alcohol compounds exhibited OAVs exceeding 1, indicating they are key aroma components making substantial contributions to the overall fruit aroma.

Among all rootstock treatments, aldehydes such as phenylacetaldehyde, octanal, nonanal, hexanal, and (Z)-3-hexenal consistently exhibited high OAVs, constituting key components of fruit aroma. Phenylacetaldehyde typically imparts floral and honey-like notes to fruit, while Octanal and Nonanal are closely associated with citrus and fruity characteristics. Significant variations in OAV levels of aldehydes were observed across different rootstock treatments.

In contrast, while C6 alcohol compounds generally exhibit higher concentrations, their OAV performance shows marked variability. For instance, (Z)-3-Hexen-1-ol and (E)-2-Hexen-1-ol yielded OAVs close to or slightly above 1 in certain rootstock treatments, yet fell below the threshold under other rootstocks. This indicates that the contribution of C6 alcohols to fruit aroma is unstable under different rootstock conditions, with their OAVs exhibiting inconsistent variations across rootstock treatments rather than representing dominant aromas.

Notably, despite their relatively low proportion in total terpenoid content, compounds such as linalool, geraniol, and α-terpineol exhibited high OAV across multiple rootstock treatments due to their extremely low olfactory thresholds. Under certain rootstock conditions, their OAV significantly surpassed that of most aldehyde compounds. Research indicates that the characteristic floral and fruity aromas of Muscat-type grapes are primarily determined by a few low-threshold terpenoids, whose sensory contributions often exceed their proportion in the total volatile compounds. The findings of this study further validate the crucial role of terpenoids in the aroma profile of ‘Shine Muscat’ fruit.

Significant differences exist in OAV distribution among fruits grafted onto different rootstocks. Among them, fruits grafted onto 101-14, 161-49, and Lot rootstocks exhibited overall higher OAVs for multiple key VOCs, particularly for terpenes and certain fruit-aroma-related aldehyde compounds. In contrast, fruits grafted onto K1 and 110R rootstocks exhibited lower OAVs for most VOCs, indicating a relatively weaker distribution of these compounds.

Further comparison revealed that fruits grown on Lot rootstock exhibited not only higher OAV for floral terpenes such as linalool and geraniol, but also maintained elevated levels of fruit-aroma-related aldehydes like phenylacetaldehyde and nonanal. This characteristic synergistic increase across multiple aroma-active compounds confers the Lot rootstock with distinct overall aroma potential.

The comprehensive OAV analysis reveals that the influence of different rootstocks on the aroma of ‘Shine Muscat’ fruit is not simply reflected in changes to the total volatile content, but rather reshapes the sensory expression of fruit aroma by regulating the OAV structure of key VOCs. In particular, the differential regulation of aroma contributions from low-threshold terpenes and fruity aldehydes constitutes a major factor underlying the aroma differences among rootstocks. This finding provides a data foundation for subsequent multivariate statistical analysis to screen rootstock types exhibiting distinct aromatic profiles.

#### 3.1.4. Multivariate Analysis Identifies Rootstocks with Distinct Aromatic Characteristics

To comprehensively evaluate the effects of different rootstocks on the aroma characteristics of ‘Shine Muscat’ fruit and identify key volatile compounds driving aroma differences among rootstocks, partial least squares–discriminant analysis (PLS-DA) was performed on grafted fruit based on odor activity value (OAV) data. This method effectively distinguishes between treatment groups by accounting for multivariate synergistic variations and has been widely applied in comparative analyses of grape and wine aroma characteristics [5,25].

The PLS-DA score plot results revealed a clear separation trend among samples from different rootstock treatments in the first two principal component spaces, indicating that rootstock type significantly influences the overall distribution of fruit aroma-active compounds (Figure 3B). Specifically, samples from rootstocks Lot, 101-14, and 161-49 were distinctly separated from others in the score plot, while rootstocks K1, 110R, and K5 showed relative clustering, indicating similar aroma profiles. This result further corroborates the objective differences in aroma expression among rootstocks observed in the preceding OAV analysis.

The statistical parameters of the model indicate that this PLS-DA model exhibits excellent fit and predictive capability. Both the R^2^X and R^2^Y values are at high levels, demonstrating the model’s strong ability to explain the relationship between the independent variable (aromatic active volatile compounds, OAV) and the dependent variable (rootstock type). Additionally, the positive Q^2^ value within an acceptable range indicates no significant overfitting, demonstrating good stability and reliability (Figure 3C). These results confirm that the PLS-DA model constructed using OAV data is suitable for discriminating the aroma characteristics of different rootstocks.

Further analysis using variable importance in projection (VIP) values (Table 2). Variables with VIP values greater than 1 are generally considered key variables that significantly contribute to the model’s classification performance [3]. In this study, a total of seven aroma-active compounds with VIP ≥ 1 were identified, primarily comprising terpenoids and certain C6 alcohols and aldehydes.

Among the screened key variables, geraniol and α-terpineol ranked highest in VIP values, indicating that these low-threshold terpenoids play a central role in distinguishing the aromatic characteristics of different rootstocks. Combined with the aforementioned OAV analysis results, it is evident that these compounds not only exhibit high OAV in certain rootstock treatments but also show significant variation across different rootstocks, making them crucial factors driving sample separation. Additionally, (Z)-3-hexen-1-ol and phenylacetaldehyde also exhibited high VIP values, indicating that green-flavored and fruity aldehydes associated with the lipoxygenase pathway similarly contribute to the formation of aroma differences among different rootstocks.

Based on the distribution characteristics of key aroma-active compounds across different rootstocks, fruits grown on the Lot rootstock exhibit simultaneously elevated OAVs for multiple compounds with high VIP scores, particularly showing distinct differences in substances like geraniol and α-terpineol. This “multiple key variables synergistically enhancing” characteristic clearly distinguishes the Lot rootstock from others in the multivariate space. In contrast, fruits grown on K1 and 110R rootstocks exhibit lower OAV for most high-VIP-value aroma compounds, causing their samples to cluster in the same region within the score plot.

The combined results of PLS-DA and VIP analyses revealed that the influence of different rootstocks on the aroma of ‘Shine Muscat’ fruit is not determined by a single volatile compound, but rather driven by the synergistic changes in multiple key aroma-active substances. The Lot rootstock exhibited significantly higher levels of several high-contributing aroma-active substances, resulting in a distinct overall aroma profile.

### 3.2. Plant Hormone Treatment

#### 3.2.1. Effects of Exogenous Plant Growth Regulators on the Morphology of Fruits Grafted onto Lot Rootstocks

**Figure 4:**
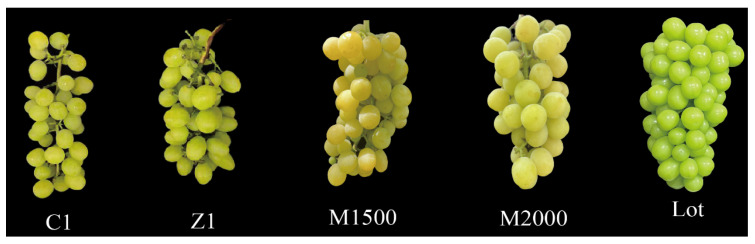
Fruit morphology after treatment with different exogenous plant growth regulators. From each group, three bunches of grapes with the same growth were selected as samples for subsequent experiments.

Based on screening rootstocks with distinct aromatic profiles, further treatments involving different exogenous plant growth regulators were applied to Lot-grafted fruits to evaluate their additional regulatory effects on fruit aroma. Fruit appearance exhibited noticeable variations under different treatments (Figure 4). GA_3_ treatments (Z1 and C1) significantly influenced fruit morphology, while MeJA treatments (M1500 and M2000) resulted in fruit coloration tending toward yellowish-green.

#### 3.2.2. Effects of Exogenous Plant Growth Regulators on the Composition of Volatile Compounds in Lot-Grafted Grape Fruits

Quantitative analysis of volatile compounds revealed that different exogenous plant growth regulator treatments significantly influenced both the total amount and composition of fruit VOCs (Table 3). Specifically, M1500 and M2000 treatments significantly increased total fruit volatile content, while Z1 and C1 treatments exhibited relatively lower total VOC levels, indicating differential effects of exogenous regulators (varying in type and concentration) on volatile synthesis. These findings suggest that exogenous plant growth regulator applications can further enhance fruit volatile metabolism levels in the context of the Lot rootstock.

Comparisons across different volatile compound categories revealed significant shifts in the relative content distribution of each category across treatment groups (Table 3). Compared to the Lot control, all exogenous plant growth regulator treatments significantly reduced fruit acid and alcohol content. Among these, acid content decreased most markedly under Z1 treatment, while alcohol content was significantly lower under M1500 and M2000 treatments compared to the control. Acids and certain alcohol compounds are typically associated with fatty or green odors. Their reduced relative content altered the distribution characteristics of volatile compounds in the fruit.

Meanwhile, ester and terpenoid compounds exhibited a general upward trend in exogenous plant growth regulator-treated groups, with the most pronounced effect observed under MeJA treatment. Compared to the Lot control, terpenoid content increased by approximately 79.7% and 71.6% under M1500 and M2000 treatments, respectively, demonstrating MeJA’s significant promotion of terpenoid accumulation. In the GA3-treated group, terpenoid content was also higher than the control, though the increase was relatively smaller. This indicates distinct differences in how various types of plant growth regulators regulate terpenoid metabolism.

At the level of specific compounds, typical aromatic monoterpenes such as linalool, geraniol, and α-terpineol showed significantly increased levels following exogenous plant growth regulator treatments, with particularly pronounced effects in the MeJA-treated group. In contrast, the Lot control group exhibited relatively low levels of these compounds. Previous studies have indicated that these terpenoids constitute the essential material basis for the floral and fruity characteristics of Muscat-type grapes, with their concentration changes often closely linked to the fruit’s aromatic profile [4]. Therefore, exogenous plant growth regulator treatment altered the distribution characteristics of fruit volatiles by increasing the accumulation of aromatic terpenoids without changing the composition of volatile compounds. Additionally, certain ester compounds exhibited a notable increase following exogenous plant growth regulator treatments, particularly in the M1500-treated group. Although ester levels remained relatively low overall in this study, their upward trend suggests that exogenous treatments may promote the accumulation of volatile compounds associated with sweet fruit aromas, thereby further enriching the fruit’s aromatic complexity.

A comprehensive analysis of the category composition and content changes in volatile compounds across different treatment groups reveals that exogenous plant growth regulators did not significantly alter the fundamental composition types of volatile compounds in ‘Shine Muscat’ fruit. Instead, they shifted the fruit’s aroma profile by regulating the relative proportions of various volatile compounds. This resulted in a transition from a structure dominated by aldehydes and alcohols toward one characterized by terpenes and esters—compounds associated with floral and fruity aromas. This finding provides foundational data support for subsequent evaluations of aroma enhancement effects across treatments using odor activity values and multivariate statistical analysis.

#### 3.2.3. OAV Analysis of Differential Aroma

**Figure 5:**
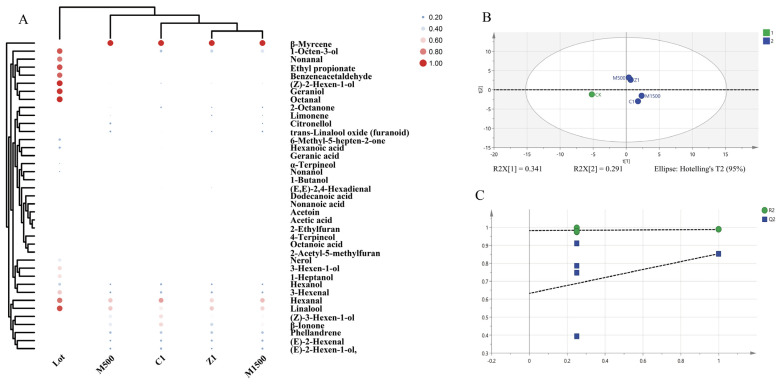
(**A**) Heat map of fruit aroma OAV clustering after treatment with exogenous plant growth regulators. Binary classification of VOCs in OAV analysis of differential aroma using PLS-DA analysis. (**B**) PLS-DA score plot for classification; (**C**) model cross-validation results. The model is based on 200 displacement tests. The data for PLS-DA was processed using only unit variance and was not log-transformed.

To further compare the effects of different exogenous plant growth regulator treatments on the sensory contribution to the aroma of ‘Shine Muscat’ fruit and to identify the treatment with the optimal aroma-enhancing effect, based on quantitative GC–MS results, the odor activity values (OAV) of volatile compounds in fruit samples from each treatment group were calculated. Combined with cluster analysis and partial least squares discriminant analysis (PLS-DA), a comprehensive evaluation of the aroma characteristics of different treatments was conducted (Figure 5). Results revealed significant differences in the composition and contribution of aroma-active compounds among treatment groups, and samples could be effectively distinguished through multivariate analysis.

OAV analysis results indicate that, under the Lot rootstock background, exogenous plant growth regulator treatments significantly altered the composition of fruit aroma-active compounds and their relative contributions. Compared to the Lot control group, the number of aroma-active compounds with OAV ≥ 1 changed across all treatment groups, and significant differences existed in the OAV distribution of key aroma-active compounds between different treatments. This suggests that exogenous regulation not only affects the content levels of volatile compounds but also alters their actual contribution to the overall fruit aroma.

Among the detected aroma-active compounds, terpenes, aldehydes, and certain C6 alcohols remained the primary constituents with elevated OAVs. Among these, aromatic monoterpenes such as α-terpineol, 4-terpineol, β-myrcene, and linalool exhibited significant OAV enhancement across multiple treatment groups, particularly under Z1 and M1500 treatments, where their OAVs were markedly higher than those of the Lot control group. Given the extremely low olfactory thresholds of these compounds, the elevation of their OAV indicates a significant enhancement of floral and fruity characteristics in the fruit at the sensory level. In contrast, OAV levels generally decreased in alcohols and acid compounds following exogenous plant growth regulator treatments, with the most pronounced effect observed under Z1 treatment. This trend aligns with the analysis of volatile compound categories, indicating that exogenous plant growth regulator treatments help mitigate the contribution of undesirable aroma compounds at the sensory level.

To visually compare the overall differences in VOCs among different treatment groups, OAV data underwent cluster analysis (Figure 5A, Table S3). Results showed that samples from each treatment group could be clearly distinguished within the cluster tree. Notably, Z1-treated samples exhibited the most pronounced separation from the Lot control group, and their cluster position revealed distinct aroma characteristics compared to other treatment groups. M1500 and C1 treatment samples were relatively close in the cluster structure, indicating some similarity in their aroma-active compound compositions. In contrast, M2000 treatment samples clustered closer to the Lot control group, suggesting a relatively limited effect on aroma enhancement.

A PLS-DA model was further constructed based on OAV data to comprehensively evaluate the effects of different exogenous plant growth regulator treatments on fruit aroma characteristics (Figure 5B). The PLS-DA score plot revealed a clear separation trend among treatment groups in the multivariate space, indicating that exogenous plant growth regulator treatments significantly altered the overall distribution of fruit aroma-active compounds. Both the R^2^ and Q^2^ parameters of the model fell within reasonable ranges, demonstrating its good explanatory and predictive capabilities (Figure 5C).

In the PLS-DA score plot (Figure 5B), the Z1-treated samples exhibited the greatest distance from the Lot control group in the principal component space, indicating the most significant change in their aroma characteristics. The M1500 and C1-treated samples also formed distinct clusters from the control group, though with slightly lower separation than Z1. The M2000-treated samples showed the least separation from the control group. This result is highly consistent with the findings from the cluster analysis.

To identify key aroma-active compounds driving flavor differences between treatments, a Variable Importance Projection (VIP) analysis was conducted on the PLS-DA model (Table 4). Results revealed that 13 aroma-active compounds exhibited VIP values exceeding 1, indicating significant contributions to treatment group differentiation. Among these, α-terpineol, 4-terpineol, cis-2-hexen-1-ol, β-myrcene, and linalool ranked highest in VIP values, suggesting that variations in these low-threshold compounds under exogenous regulation are key factors driving sample separation.

The integrated results from OAV, cluster analysis, and PLS-DA reveal significant differences in aroma enhancement effects among different exogenous plant growth regulator treatments. Among them, the Z1 treatment exhibited optimal characteristics in overall OAV levels, the number of key aroma-active compounds, and the degree of separation in multivariate analysis. M1500 and C1 followed as the next best treatments, while the M2000 treatment showed relatively weaker aroma enhancement effects. These findings indicate that under the Lot rootstock background, Z1 represents the most promising exogenous plant growth regulator treatment, providing clear experimental evidence for further aroma regulation studies.

#### 3.2.4. Analysis of Differences in Aroma Composition

Based on the OAV analysis of volatile aroma compounds across different treatment groups, orthogonal partial least squares discriminant analysis (PLS-DA) was performed (Figure 5), using differential OAVs as dependent variables and exogenous plant growth regulator treatments as independent variables. The PLS-DA model effectively distinguished the mature grape samples among the treatment groups. For the exogenous regulator treatment dataset, the PLS-DA model achieved an R^2^X (cumulative explained variance of predictors) of 0.631, an R^2^Y (cumulative explained variance of the response) of 0.987, and a Q^2^ (predictive ability) of 0.853. These values indicate that the model has strong explanatory and predictive power, supporting reliable differentiation of aroma characteristics influenced by various PGR treatments.

Using a variable importance in projection (VIP) threshold greater than 1 [26], a total of 13 volatile organic compounds (VOCs) with high OAVs were identified as key aroma contributors under exogenous plant growth regulator treatments (Table 4). Among these, VOCs with both high VIP scores and low odor thresholds were considered the main contributors to the fruit aroma profile. Specifically, lauric acid showed a high OAV in the M1500 group; cis-2-ethen-1-ol had elevated OAVs in the C1 and Z1 groups; 4-terpineol maintained high OAVs across all treatment groups; and α-terpineol consistently had a high OAV following treatments.

When comparing the aroma intensities based on OAV across treatment groups, the relative increases followed this order: Z1 > M1500 > C1 > M2000, with approximately 8-fold, 7-fold, 5-fold, and 1.5-fold increases over the control (Lot), respectively. These results indicate that the Z1 treatment caused the most significant increase in grape aroma among all tested conditions.

### 3.3. Comparison of Rootstocks and Exogenous Plant Growth Regulators

Comparative analysis of fruit aroma composition under Lot rootstock treatment and Z1 exogenous plant growth regulator treatment (Table 5) revealed that, in terms of volatile compound structure, both treatments detected the same categories of volatiles in fruit, including aldehydes, alcohols, terpenes, acids, esters, and ketones. This indicates that neither regulatory approach altered the fundamental composition types of volatile compounds in ‘Shine Muscat’ fruit. However, significant differences were observed in the relative content and proportional distribution of volatile compounds across different categories.

Under the Lot rootstock treatment, aldehydes and alcohols remained the primary components of fruit volatiles, while terpenes and esters accounted for relatively lower proportions. However, their content was significantly higher than in other rootstock treatments, establishing a relatively stable baseline aromatic metabolic profile. These findings indicate that rootstock selection primarily influences the overall aromatic potential of fruit by regulating the accumulation levels of fundamental volatiles.

In contrast, under the Lot rootstock background, the application of the exogenous plant growth regulator Z1 treatment resulted in further alterations to the proportion structure of fruit volatiles. Compared to the Lot control, Z1 treatment significantly reduced the relative content of acids and certain alcohols while increasing the proportion of terpenes and ketones. This resulted in a trend toward enrichment of floral and fruity-related compounds in the volatile profile. This indicates that exogenous plant growth regulators further optimize the fruit aroma structure within the basal metabolic framework provided by the rootstock.

Comparing from the perspective of Odor Activity Value (OAV), fruits grown on the Lot rootstock exhibit OAV ≥ 1 for multiple aroma-active compounds, particularly C6 alcohols and certain aromatic terpenes, making significant contributions to the fundamental sensory expression of fruit aroma. Under Z1 treatment, the number of aroma-active compounds with OAV ≥ 1 further increased. The OAVs for multiple low-threshold terpenes and ketones were significantly higher than those in the Lot control group, indicating stronger contributions to floral and fruity sensory perceptions.

Further comparative analysis of key aroma-active compounds revealed that the Lot rootstock primarily enhanced compounds associated with freshness and fruity notes, such as (Z)-3-hexen-1-ol and phenylacetaldehyde. Meanwhile, the Z1 treatment significantly increased the aromatic contribution of terpenoid compounds like α-terpineol, 4-terpineol, and β-myrcene. This indicates that the two regulatory strategies exhibit distinct focuses in shaping fruit aroma.

Based on the aforementioned multivariate statistical analysis results derived from OAV data, it is evident that rootstock selection alone can significantly influence fruit aroma characteristics. Furthermore, under the influence of aroma-advantaged rootstocks, exogenous plant growth regulator treatments can amplify aroma differences between samples, demonstrating a synergistic enhancement effect. In summary, rootstock selection and exogenous plant growth regulator treatment play distinct yet complementary roles in regulating the aroma of ‘Shine Muscat’ fruit. Rootstock selection primarily determines the fundamental metabolic background and potential expression capacity of fruit aroma, while exogenous regulation finely tunes key aromatic compounds on this foundation, thereby further enhancing the floral and fruity characteristics of the fruit.

### 3.4. Antifungal Activity Evaluation

#### 3.4.1. Morphological Characterization

**Figure 6:**
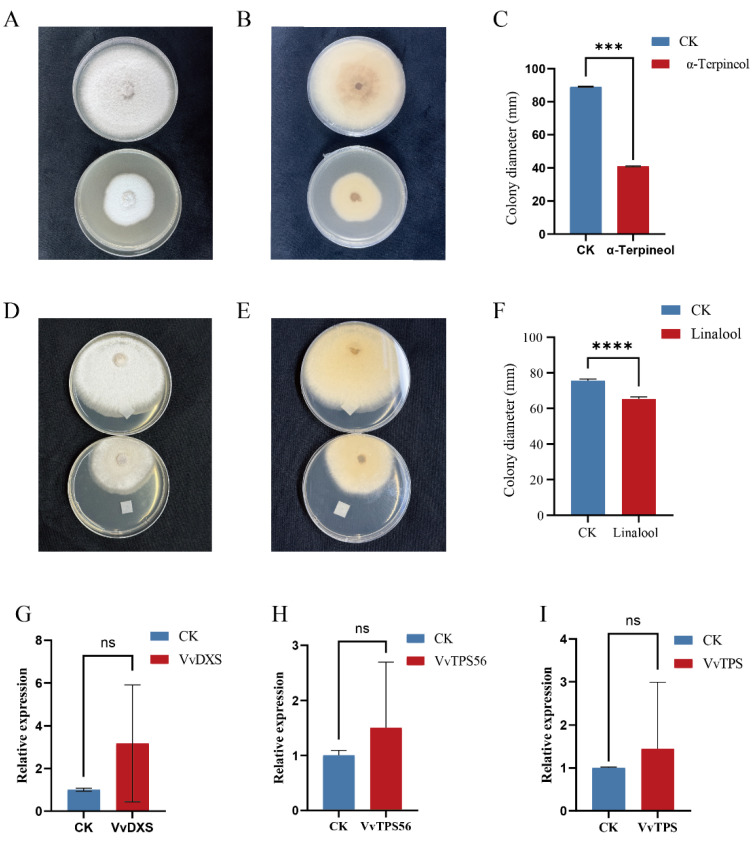
Cultivation Characteristics of *Colletotrichum fructicola*. (**A**) Upper colony surface. (**B**) lower colony surface. (**C**) Statistical bar chart showing the effect of α-Terpineol on colony size of *Colletotrichum fructicola* after 7 days of inoculation in PDA medium. Changes in Major Terpenoid Compound Enzyme Genes in Leaves of ‘Shine Muscat’ Grapes After Inoculation with *Colletotrichum fructicola.* (**D**) Upper colony surface. (**E**) lower colony surface. (**F**) Effect of Linalool on in vitro conidial germination. (**G**) Changes in *VvDXS* expression in leaves after inoculation with *Colletotrichum fructicola*. (**H**) Changes after inoculation of linalool synthase. (**I**) Changes in α-Terpineol Synthase. Vertical bars indicate mean ± SD (In Figure (**C**,**F**), n = 10; in Figure (**G**–**I**), *n* = 3), and different * indicate significant differences between different kinds of substances (one-way ANOVA, *p* < 0.05) and “ns” statistically significant difference (*p* > 0.05).

When cultured on potato dextrose agar (PDA), the colonies appeared white (Figure 6A,B). After 7 days of cultivation, cDNA was extracted from the isolates, and *ITS*, *Actin*, and *GAPDH* were amplified and sequenced; the resulting sequences have been deposited in GenBank (Table 6). The BLASTn alignment results show that the *ITS*, *Actin*, and *GAPDH* sequences of this strain are 98–99% identical to those of *Colletotrichum fructicola*.

#### 3.4.2. Effects of α-Terpineol and Linalool on the Mycelial Growth

To simultaneously investigate whether the application of exogenous plant growth regulators and grafting onto rootstocks improves the antibacterial properties of grapevines, α-Terpineol—a major terpenoid compound common to both Lot and Z1 treatments—was selected. The methodology was adapted from previous research [20]. The effects of α-Terpineol on the mycelial growth of *Colletotrichum fructicola* on PDA are shown in Figure 6A. As the concentration of α-Terpineol increased, mycelial growth showed inhibition (Figure 6A,B). After 7 days of incubation, the control group had a colony diameter of 89 mm (Figure 6C). Notably, the α-Terpineol concentration reached 55 μL·L^−1^, and mycelial growth of the fruit rot pathogen was inhibited, with colony diameter reduced to 41 mm (Figure 6C). This result indicates that α-Terpineol vapor effectively suppresses the growth of the fruit rot pathogen.

Linalool reduced the colony diameter of *Colletotrichum fructicola* through its volatility (Figure 6D,E). In the untreated control group, the average colony diameter reached 75.5 mm after 7 days of inoculation. In the Linalool-treated group, the colony diameter decreased by 13.9% (Figure 6F). These results indicate that under certain concentrations of linalool, the growth of *Colletotrichum fructicola* colonies is significantly inhibited. Colony inhibition experiments demonstrate that terpenoid compounds such as linalool and α-terpineol can inhibit the growth process of *Colletotrichum fructicola* to a certain extent.

#### 3.4.3. Effects of *Colletotrichum fructicola* on Grapes

Results revealed a significant increase in gene expression of *DXS,* the initial enzyme in monoterpene biosynthesis (Figure 6G). Compared to the control group, *DXS* gene expression levels increased threefold. Concurrently, the expression levels of Linalool Synthase (*TPS56*) and α-Terpineol Synthase (*TPS*) also showed improvements of varying degrees (Figure 6H,I). Specifically, *TPS56* and *TPS* expression levels increased by 50% and 40%, respectively, compared to the control group. The results indicate that following *Colletotrichum fructicola* infection, grape leaves exhibit increased levels of linalool and α-terpineol among terpenoid compounds to counteract external stress.

## 4. Discussion

This study systematically evaluated the effects of different rootstock combinations and exogenous plant growth regulators (GA_3_ and MeJA) on the composition and aroma activity contribution of volatile aroma compounds in table grape fruits. The research aimed to clarify the role of rootstock selection and hormonal regulation in aroma formation and provide a scientific basis for aroma-directed cultivation management.

Research findings indicate that different rootstocks exert a significant influence on the composition and content of volatile aromatic compounds in fruit. Although the types of volatile compounds detected in fruits across treatments were generally consistent, the relative abundance of various aromatic substances differed markedly under different rootstock backgrounds, particularly in the accumulation levels of C6 compounds, alcohols, and certain esters. This indicates that rootstocks do not merely influence aroma formation indirectly through effects on fruit growth or ripeness, but can actively shape the characteristic aroma profile of the fruit to a certain extent. Further analysis of odor activity values revealed differences in the contribution of key aroma-active compounds among rootstock treatments. These variations likely represent a major factor underlying the overall differences in perceived fruit aroma.

Among various rootstock treatments, certain rootstocks not only maintain stable basic physicochemical qualities in fruit but also promote the accumulation of compounds with higher aromatic contribution in fruit, demonstrating relatively superior aromatic potential. This finding provides a basis for optimizing aromatic quality through rational rootstock selection in production practice, while also validating the research hypothesis stated in the introduction that “rootstock serves as a key agronomic factor for aroma regulation.”

The results of this study indicate that the Z1 treatment (15.25 mg·L^−1^ GA_3_) significantly outperformed the C1 treatment (22.5 mg·L^−1^ GA_3_) in promoting terpenoid accumulation, demonstrating that GA_3_ exerts a pronounced dose-dependent regulatory effect on grape aroma metabolism. This phenomenon may be related to the “low-dose stimulation—high-dose inhibition” (hormesis) response pattern of plant hormones on secondary metabolism. Appropriate exogenous GA_3_ can effectively promote cell division and activate certain secondary metabolic pathways, thereby improving fruit quality and enhancing the accumulation of aromatic compounds to a certain extent [27]. However, when GA_3_ concentrations are excessively high (as in the C1 treatment), plants allocate more energy and nutrients to primary vegetative growth processes. This leads to phenomena such as excessive cell elongation, thickened fruit peel, or petiole lignification [28], thereby creating competition for resources with secondary metabolism (aroma compound synthesis). Additionally, high concentrations of GA_3_ may cause excessive fruit enlargement, delay sugar accumulation and the ripening process, thereby indirectly inhibiting the biosynthesis of terpenoid aromatic compounds [29].

Notably, the regulatory effects of GA_3_ and MeJA were both influenced by rootstock background. Under identical hormone treatments, fruit aroma responses varied across different rootstock combinations, suggesting potential synergistic or limiting interactions between rootstock and exogenous regulators. This finding further supports the perspective outlined in the introduction: evaluating the aroma effects of exogenous regulators or rootstocks in isolation fails to fully reflect regulatory outcomes under real-world production conditions. Instead, their combined effects hold greater practical significance. Unlike previous studies that primarily focused on the antibacterial activity of individual volatile compounds or changes in resistance gene expression under pathogen infection conditions, this research integrates cultivation practices regulating fruit aroma metabolism, the antibacterial function of key terpenoid aroma compounds, and the corresponding expression responses of synthesis genes. It reveals, at an integrative level, the potential link between aroma quality formation and disease defense mechanisms. Results indicate that regulating terpenoid metabolism through rootstock grafting and exogenous plant growth regulators not only enhances fruit aroma quality but also enhances the ability of grape leaves to resist pathogens under in vitro conditions. α-Terpineol and Linalool consistently exhibit high OAVs and VIP scores, indicating their pivotal role in aroma differentiation. Previous studies have also demonstrated that linalool plays a significant role in plant defense against biotic and abiotic stresses [30,31]. Individual plants often rely on the synergistic action of multiple volatiles for natural defense. The overall synergistic effects of volatile compounds will serve as a direction for future research.

Overall, this study demonstrates that rootstock grafting plays a fundamental role in shaping the volatile aroma characteristics of table grapes and determines the efficacy of exogenous GA_3_ and MeJA in regulating aroma metabolism. By integrating aroma metabolism analysis with antibacterial experiments and related synthetic gene expression, the findings further confirm that cultivation practices targeting terpenoid metabolism not only enhance fruit aroma quality but may also simultaneously boost disease resistance in ‘Shine Muscat’. This research provides scientific support for aroma-directed grape cultivation strategies that simultaneously advance quality improvement and disease control.

## 5. Conclusions

The results of this study indicate that 11 commonly used rootstocks significantly altered the accumulation profiles of volatile compounds in ‘Shine Muscat’ fruit, particularly exerting differential regulatory effects on key terpenes such as linalool and geraniol, as well as C6 aldehydes and alcohols [11,32,33]. Comprehensive evaluation based on odor activity value (OAV) and partial least squares discriminant analysis (PLS-DA) indicates that Lot rootstock provides the optimal contribution to key aroma compounds in grafted fruit, making it a suitable rootstock for enhancing fruit aroma potential. Under the Lot rootstock, foliar application of 15.25 mg L^−1^ GA_3_ (Z1 treatment) and 1500 μmol L^−1^ MeJA (M1500 treatment) both effectively reduced the sensory contribution of acids and certain alcohols while significantly promoting the accumulation of terpenes (α-terpineol, 4-terpineol) and ketones associated with floral and fruity aromas, thereby optimizing the aroma profile [34,35]. The rootstock primarily establishes the foundational framework for fruit aroma metabolism, while exogenous regulators perform precise, targeted modulation of key aromatic compounds within this framework. The combination of Lot rootstock grafting with Z1 treatment (15.25 mg L^−1^ GA_3_) demonstrated the most pronounced aroma enhancement, achieving synergistic improvement in overall fruit aroma quality. This study confirms that under specific cultivation conditions, the combined intervention of rootstock selection and exogenous hormones not only reshapes the aroma metabolic network of “Shine Musca” grapes but also demonstrates significant potential for synergistic interaction with the plant defense system. This conclusion rests upon two key lines of evidence: First, in vitro antibacterial assays confirmed the inhibitory effects of core aroma compounds (α-Terpineol and Linalool) against the grape anthracnose pathogen *Colletotrichum fructicola* [36]. Second, pathogen infection effectively activated a surge in expression of core terpenoid synthesis genes (*DXS*, *TPS56*, and *TPS*) within leaf tissues. This cross-tissue physiological response suggests a valuable theoretical framework: MEP pathway upregulation induced by agronomic practices may simultaneously drive fruit aroma compound accumulation while conferring enhanced defensive potential to nutrient organs. However, evidence ranging from in vitro antibacterial activity to leaf transcriptional responses does not directly equate to the establishment of whole-plant systemic immunity. To translate this “dual-targeting quality and resistance” cultivation concept into agricultural practice, future efforts must overcome current tissue and spatial limitations. Conducting in vivo fruit disease-centered validation studies across multidimensional ecological regions and multi-year orchard environments will be essential for confirming this strategy’s field efficacy.

## Figures and Tables

**Table 1:** Each test treatment concentration and treatment methods.

Treatment	Groups	Concentration
Graft	1103P, 3309M, S04, K3, K5, 110R, 8B, K1, 101-14, Lot, 161-49	1103 Paulsen, 3309 Millardet, S04, Kangzhen No.3, Kangzhen No.5, 110 Richter, Telekki 8B, Kangzhen No.1, Millardet Grasset 101-14, Rupestris du Lot, Couderc 161-49
Spray	C1	22.5 mg L^−1^ GA_3_
	Z1	15.25 mg L^−1^ GA_3_
	M1500	1500 μmol L^−1^ MeJA
	M2000	2000 μmol L^−1^ MeJA

**Table 2:** Differences in aroma compounds in grape berries grafted on various rootstocks.

Substance	Thresholds	VIP
α-Citral	0.032	1.67136
α-Terpineol	1.2	1.63945
6-Methyl-5-hepten-2-one	0.068	1.39869
Octanoic acid	3	1.11711
(Z)-3-Hexen-1-ol	0.013	1.07564
Nonanol	1	1.05815
Geraniol	0.0011	1.02115

**Table 3:** Content of each substance and total amount in each treatment group.

	Acids µg/L	Alcohol µg/L	Aldehyde µg/L	Lipids µg/L	Terpenes µg/L	Total µg/L
Z1	0.88 ± 0.07 ^c^	13.26 ± 1.05 ^c^	52.24 ± 4.18 ^c^	37.66 ± 3.02 ^a^	19.37 ± 1.55 ^c^	123.41 ± 9.87 ^c^
C1	2.07 ± 0.18 ^b^	16.12 ± 1.29 ^c^	56.83 ± 4.55 ^c^	38.56 ± 3.08 ^a^	13.24 ± 1.06 ^c^	126.82 ± 10.15 ^c^
M1500	1.71 ± 0.14 ^bc^	11.32 ± 0.91 ^c^	73.46 ± 5.88 ^b^	37.75 ± 3.02 ^a^	48.07 ± 3.85 ^a^	172.31 ± 13.78 ^a^
M2000	2.42 ± 0.19 ^b^	12.49 ± 1.00 ^c^	77.27 ± 6.18 ^b^	38.48 ± 3.08 ^a^	34.20 ± 2.74 ^b^	164.86 ± 13.19 ^a^
Lot	5.70 ± 0.46 ^a^	59.59 ± 4.77 ^a^	77.27 ± 6.18 ^b^	8.40 ± 0.67 ^a^	9.71 ± 0.78 ^c^	134.79 ± 10.78 ^b^

Values are presented as mean ± SD (*n* = 3). Different lowercase letters within the same column indicate significant differences among treatments at *p* < 0.05 according to one-way ANOVA followed by Tukey’s multiple comparison test.

**Table 4:** Differential aroma substances in grape berries sprayed with different exogenous plant growth regulators.

Substance	Cas	VIP
Dodecanoic acid	143-07-7	1.67922
(Z)-2-Hexen-1-ol	928-94-9	1.42585
Nonanol	143-08-8	1.41194
α-Terpineol	98-55-5	1.31358
4-Terpineol	562-74-3	1.3045
Hexanal	66-25-1	1.29828
Phellandrene	99-83-2	1.29182
(E)-2-Hexen-1-ol,	928-95-0	1.11812
Acetic acid	64-19-7	1.11141
2-Ethylfuran	3208-16-0	1.05252
3-Hexen-1-ol	544-12-7	1.02773
(Z)-3-Hexen-1-ol	928-96-1	1.02225
trans-Linalool oxide (furanoid)	143-07-7	1.67922

**Table 5:** Comparison of fruit aroma substances under two treatments.

	Substance	Lot	Z1
Acids	Octanoic acid	0.80027	0.39152
	Hexanoic acid	4.47546	0.27704
	Acetic acid	-	0.21126
	Nonanoic acid	0.41889	-
Alcohols	Nonanol	1.15536	0.07369
	1-Octen-3-ol	1.15536	0.08541
	3-Hexen-1-ol	-	0.04237
	1-Butanol	-	0.10714
	(Z)-2-Hexen-1-ol	-	0.38687
	(E)-2-Hexen-1-ol	16.65962	0.92273
	(Z)-3-Hexen-1-ol	20.13638	2.97670
	1-Heptanol	0.33862	-
	Hexanol	20.14248	8.66505
Aldehydes	Hexanal	32.65962	46.37704
	(E, E)-2,4-Hexadienal	-	0.52289
	3-Ethyl-benzaldehyde	-	2.22112
	Isophthalaldehyde	-	0.41854
	(E)-2-Hexenal	-	0.91448
	3-Hexenal	15.14248	1.78839
	Benzeneacetaldehyde	1.19775	-
	Octanal	1.19775	-
	Nonanal	1.19775	-
Esters	Ethyl propionate	8.39733	-
Ketones	6-Methyl-5-hepten-2-one	0.41889	-
	2-Octanone	-	0.48534
	Acetoin	-	0.28640
	β-Ionone	-	0.21805
Others	2-Ethylfuran	-	0.03274
	2-Acetyl-5-methylfuran	-	0.10677
Terpenes	Geraniol	1.48404	-
	Nerol	1.25456	-
	Linalool	5.52513	5.11586
	α-Terpineol	1.02851	0.56701
	β-Myrcene	-	74.21959
	Limonene	-	0.54132
	trans-Linalool oxide (furanoid)	-	0.57334
	Geranic acid	-	0.52610
	4-Terpineol	-	0.18237
	1-(4-Ethylphenyl)-ethanone	-	0.06427
	Phellandrene	-	1.32803

**Table 6:** List of *Colletotrichum fructicola* strains in this study.

Species	Host	Country	GeneBank No.		
			ITS	ACT	GAPDH
*C. fructicola*	*Vitis vinifera*	China	PZ158858	PZ189200	PZ189201

## Data Availability

The original contributions presented in this study are included in the article/Supplementary Materials.

## Supplementary Materials

The following supporting information can be downloaded at: https://www.mdpi.com/article/10.3390/life16040567/s1.
